# Complete genome sequence of *Sphingobacterium thalpophilum* BAA-1094

**DOI:** 10.1128/mra.00423-24

**Published:** 2024-10-14

**Authors:** Barbara I. Adaikpoh, Alessandra S. Eustáquio

**Affiliations:** 1 Department of Pharmaceutical Sciences, College of Pharmacy, University of Illinois at Chicago, Chicago, Illinois, USA; 2 Center for Biomolecular Sciences, College of Pharmacy, University of Illinois at Chicago, Chicago, Illinois, USA; Indiana University, Bloomington, Bloomington, Indiana, USA

**Keywords:** *Sphingobacterium*, complete genome sequence

## Abstract

We report the complete genome of *Sphingobacterium thalpophilum* BAA-1094 obtained from the American Type Culture Collection. The genome has one circular chromosome (5.7 Mbp). The rather low average nucleotide identity of 76.5% to the type strain *Sphingobacterium thalpophilum* DSM 11723 suggests the need for reclassification.

## ANNOUNCEMENT

The *Sphingobacterium* genus is associated with suppressing fungal phytopathogens (1–3), producing enzymes for organic polymer biodegradation (1, 4–8) and natural product biosynthesis (9). We report the genome of *Sphingobacterium thalpophilum* BAA-1094 obtained from the American Type Culture Collection (Virginia, USA) as a water isolate. BAA-1094 is routinely cultured in trypticase soy broth (TSB) at 30°C.

Genomic DNA from harvested cells grown in 5-mL TSB for 18 h at 200 rpm, was extracted with the GenElute Bacteria Genomic DNA kit (Sigma-Aldrich NA2120) according to the manufacturer’s instructions. Library preparation and sequencing were carried out at SeqCenter (Philadelphia, USA). Nanopore sequencing libraries were prepared using the Native Barcoding Ligation Sequencing Kit 24 V14 with the NEBNext Companion Module according to Oxford Nanopore Technologies’ specifications (Oxford, UK). DNA fragmentation or size selection was not performed. The library was sequenced on a MinION Mk1B sequencer using the R10.4.1 flow cell. Guppy (v.6.4.6, dna_r10.4.1_e8.2_400bps_modbases_5mc_cg_sup.cfg) was used for base calling, demultiplexing, and adapter removal. A total of 1,647,523 reads were obtained. Illumina sequencing libraries were prepared using the Illumina DNA Prep Kit (California, USA) and custom IDT 10-bp unique dual indices with a target insert size of 320 bp. Sequencing was performed with a NovaSeq 6000 sequencer, employing 2 × 151-bp paired-end reads to obtain 4,276,158 reads. Quality control and adapter trimming were performed with bcl-convert (v.4.1.5).

Nanopore reads trimmed with Porechop (v.2.4) were *de novo* genome-assembled with Flye (v.2.9.2, parameters: --asm-coverage 50 --genome-size 6000000 --nano-hq) (10). A coverage of 272.3× and N50 of 5,752,567 were obtained. Hybrid polishing with Illumina reads was done with Pilon (v.1.24) (11). The assembly was circularized and rotated to have its start position set to *dnaA* with Circulator (v.1.5.5) (12) and annotated with the NCBI Prokaryotic Genome Annotation Pipeline (13). Taxonomy was confirmed with the Type Strain Genome Server (14). Default parameters were used for all software except where otherwise noted.

**Fig 1:**
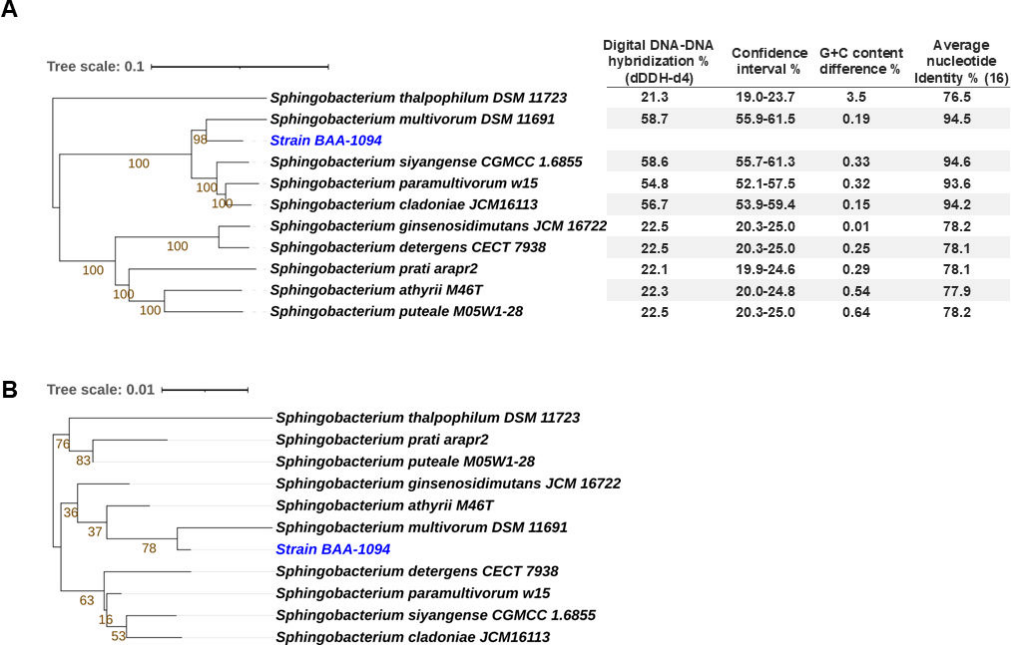
Phylogenetic tree for *Sphingobacterium thalpophilum* BAA-1094 using the Type Strain Genome Server (14). From automatically selected closest type strains, trees were inferred from GBDP distances calculated from genome sequences (**A**) or 16S rDNA sequences (**B**). Branch lengths are scaled in terms of GBDP formula d5. The numbers above the branches are GBDP pseudobootstrap values >60% from 100 replications. BAA-1094 is presented in blue font (16).

The final genome sequence consists of one circular 5,752,567-bp chromosome with 40.1% G+C content containing 5,088 genes, including 4,949 protein-coding sequences, 82 tRNAs, 7 operons (containing 5S, 16S, and 23S rRNAs), and 33 pseudogenes. BAA-1094 had a 76.5% average nucleotide identity (ANI) to the type strain *Sphingobacterium thalpophilum* DSM 11723 and clustered rather with *Sphingobacterium multivorum* DSM 1169 (94.5% ANI) (Fig. 1). Thus, reclassification may be necessary in the future (15).

## Data Availability

The assembled genome sequence of *Sphingobacterium thalpophilum* BAA-1094 is deposited at GenBank under accession no. CP151087. This paper describes the first version. Raw sequence reads were submitted to the SRA under the BioSample accession number SAMN40761716 and BioProject accession number PRJNA1096750.
